# Combination Therapy of Cefiderocol and Polymyxin B Against *Pseudomonas aeruginosa* Keratitis Isolates In Vitro

**DOI:** 10.1167/tvst.14.11.7

**Published:** 2025-11-12

**Authors:** Eric G. Romanowski, Emily K. Young, Sonya M. Mumper, Kathleen A. Yates, Rachel A. F. Wozniak, Michael E. Zegans, Robert M. Q. Shanks

**Affiliations:** 1 Department of Ophthalmology, University of Pittsburgh, Pittsburgh, PA, USA; 2 Department of Ophthalmology, University of Rochester Medical Center, Rochester, NY, USA; 3 Department of Surgery, Geisel School of Medicine at Dartmouth, Hanover, NH, USA

**Keywords:** microbial keratitis, *Pseudomonas aeruginosa* (*P. aeruginosa*), cefiderocol (FDC), antibiotic synergy, quorum sensing

## Abstract

**Purpose:**

Antibiotic combination therapy is commonly used for empiric treatment of microbial keratitis, a potentially blinding infection of the cornea. *Pseudomonas aeruginosa* (*P. aeruginosa*) is a major cause of severe keratitis, and the 2023 outbreak linked to artificial tears involved a strain resistant to aminoglycosides and fluoroquinolones, resulting in widespread vision loss. Despite various proposed treatments, no consensus exists for management of keratitis caused by the outbreak strain. The purpose of this study was to evaluate the efficacy of cefiderocol (FDC) combined with polymyxin B (PB) against ocular *P. aeruginosa* keratitis isolates, including extensively drug-resistant (XDR) strains from the 2023 artificial tear-associated outbreak.

**Methods:**

Time-kill assays and epsilometer (E-test) synergy testing were performed on a panel of keratitis isolates. Genetic analysis was conducted on mutants with reduced FDC susceptibility.

**Results:**

Time-kill assays demonstrated synergy for two XDR outbreak isolates (*P* < 0.05), whereas the E-test used across diverse keratitis isolates showed additive to indifferent interactions. Quorum-sensing-deficient, sheen-positive strains showed reduced responsiveness (*P* < 0.01). Non-antagonistic interactions were observed for *Serratia marcescens* (*S. marcescens*) and *Staphylococcus aureus* (*S. aureus*) keratitis isolates, with synergy for some *S. marcescens* strains. Mutants with reduced FDC susceptibility harbored mutations in *baeS* and *fepA*, yet remained responsive to the antibiotic combination.

**Conclusions:**

FDC combined with PB demonstrates promising activity against drug-resistant Gram-negative keratitis pathogens, including XDR *P. aeruginosa*.

**Translational Relevance:**

These findings support the further development of FDC with PB as a candidate therapy for drug-resistant ocular infections.

## Introduction

The Gram-negative bacterium *Pseudomonas aeruginosa* (*P. aeruginosa*) is a common cause of vision-threatening microbial keratitis.^1^^–^^6^ However, given the aggressive and rapid nature of infectious keratitis regardless of etiology, initial treatment of suspected bacterial keratitis is empiric, and typically involves topical antibiotics effective against Gram-negative and -positive organisms, such as fluoroquinolone monotherapy or a combination of compounded vancomycin and tobramycin.^7^ This approach is generally effective in eradicating bacterial pathogens from the cornea. Although antibiotic resistance among keratitis isolates of *P. aeruginosa* varies by region, it remains relatively uncommon in countries like Australia and the United States.^8^^,^^9^ However, infections caused by drug-resistant isolates have poorer clinical outcomes and incur higher costs.^3^^,^^10^^–^^13^

In 2023 to 2024, an outbreak of extensively drug resistant (XDR) *P. aeruginosa* occurred in 18 states in the United States, linked to preservative-free, multidose artificial tear drops.^14^^–^^16^ The outbreak isolates were resistant to all antibiotics used for treatment of ocular infections that were tested by the Centers for Disease Control and Prevention (CDC), including amikacin, cefepime, ceftazidime (CAZ), ciprofloxacin, gentamicin, meropenem, levofloxacin, and tobramycin, and resulted in numerous cases of blindness and death.^15^^,^^16^ The outbreak isolates were of sequence type 1203 (ST1203), a sequence type not previously found in the United States^17^^,^^18^ and harbored numerous antibiotic resistance determinants (20 perfect hits determined by the Comprehensive Antibiotic Resistance Database)^17^ including those for several carbapenemases. These include the Verona integron-mediated metallo-β-lactamase and the Guiana extended-spectrum β-lactamase.^15^ The CDC reported that the outbreak strains were susceptible only to cefiderocol (FDC), a siderophore-cephalosporin antibiotic, and had intermediate susceptibility to colistin (polymyxin E).^19^ Alarmingly, beyond human infections, ST1203 bacteria were determined to cause eye infections in pets in the United States.^20^

In response, our group previously investigated the potential of FDC as a topical treatment for keratitis. Data indicated that all tested Gram-negative ocular bacterial isolates, including *P. aeruginosa*, *Klebsiella* species, *Moraxella* species, *Serratia marcescens*, and others (*n* = >100) were susceptible to FDC based on systemic break points in vitro. Furthermore, FDC demonstrated bactericidal activity against keratitis *P. aeruginosa* isolates, including an outbreak isolate, in an animal model of keratitis.^21^^,^^22^

Recent studies have proposed antibiotic combinations for the treatment of bacterial keratitis aiming to broaden coverage, reduce development of antibiotic resistance, and enhance efficacy through additive or synergistic interactions.^23^^–^^28^ Based on this rationale, we evaluated polymyxin B (PB) in combination with FDC. PB has been found to enhance the function of other antimicrobials, by disrupting the outer membrane of Gram-negative bacteria thereby facilitating antibiotic access to intracellular targets,^29^^,^^30^ and is often used in ophthalmic formulations, frequently in combination with other antimicrobials like trimethoprim.^26^^,^^27^ Importantly, PB shares a mechanism of action with colistin to which the 2023 outbreak strain had intermediate susceptibility.

In this study, no antagonism was observed between FDC and PB in vitro. Further, approximately 20% of our keratitis isolates exhibited a sheen colony phenotype, often associated with mutations in the quorum-sensing regulator *lasR*, which correlates with worse clinical outcomes in ocular and pulmonary infections.^31^^–^^34^ Keratitis isolates of *Serratia marcescens* (*S. marcescens*) and *Staphylococcus aureus* (*S. aureus*) were also tested with the drug combination because they have limited susceptibility to one or both of the antibiotics individually. Finally, we generated strains with lower FDC susceptibility to determine whether resistance affected the interaction between FDC and PB. Together, our findings suggest that FDC and PB have synergistic effects versus the tested XDR outbreak isolates and, at minimum, non-antagonistic effects against other keratitis isolates.

## Methods

### Bacterial Strains, Growth, and Minimum Inhibitory Concentration Testing

Deidentified keratitis isolates of *P. aeruginosa*, *S. marcescens*, and *S. aureus* were obtained from the Charles T. Campbell Ophthalmic Microbiology Laboratory (University of Pittsburgh) or obtained from the CDC. Bacterial strains from frozen stocks were plated onto trypticase-soy agar with 5% sheep's red blood cells (BD BBL, blood agar) and incubated at 37°C for 18 to 20 hours. The sheen phenotype was established visually.

Minimal inhibitory concentrations (MICs) for FDC, CAZ, and PB were performed using MIC epsilometer test strips (E-tests; LIOFILCHEM, Waltham, MA) on Muller-Hinton II agar, as previously described.^22^ To measure combinatorial effect of the antibiotics, the E-tests on inoculated plates were at a 90-degree angle, intersecting at the previously determined MIC values.^35^ Plates were incubated for 24 hours at 37°C, and MICs were interpreted following the manufacturer's guidelines. The fractional inhibitory concentration index (FICI) was calculated as follows: FICI = (MIC_A+B_/MIC_A_) + (MIC_B+A_/MIC_B_). The FICIs were interpreted using the following criteria: ≤0.5 synergy, >0.5 to 1 additive, >1 to 4 indifference, and >4 antagonism.

### Time-Kill Analysis

FDC (FETROJA, Shionogi Inc., Florham Park, NJ) was reconstituted from a 1*g* vial according to the manufacturer instructions and prior protocols.^21^^,^^36^ A 50 mg/mL solution was prepared, aliquoted, and stored at −80°C. For each time-kill assay, a fresh aliquot was thawed and diluted in iron-depleted, cation-adjusted, Mueller-Hinton broth (ID-CA-MHB) prepared according to published methods.^37^

PB sulfate (EMC Millipore Corp., Cat. No. 5291) was dissolved in water at 1 mg/mL, filter sterilized, and stored at 4°C. The FDC and the PB concentrations were diluted in ID-CA-MHB the day prior to the time-kill assays.

Bacterial suspensions of *P. aeruginosa* were adjusted to a 0.5 McFarland standard (∼10^8^ CFU/mL) in 1.0 mL of ID-CA-MHB. From these, 3.0 µL was inoculated into 1 mL of antibiotic-containing or control media to yield approximately 5 × 10^5^ CFU/mL. Tubes were vortexed for 30 seconds and incubated at 37°C. Viable counts were prepared at 0, 1, 2, 4, 6, 8, and 24 hours. Resulting aliquots were serially diluted in MHB, plated on blood agar, and incubated at 32°C overnight. Colony counts were enumerated after incubation.

Each strain and antibiotic condition were tested in at least three independent experiments. Antibiotic concentrations were 2 times the MIC of FDC and 0.333 times the MIC of PB, as determined by E-tests. These values relative to the MIC were based on the effectiveness of the antibiotics and combinations in preliminary trials.

The limit of detection was 100 CFU/mL. For statistical analysis, counts below this threshold were assigned a value of 10 CFU/mL. The CFU/mL values were Log_10_-transformed, and mean Log_10_ ± SD CFU/mL was calculated for each group.

Antibiotic interaction outcomes were classified based on established criteria.^35^ Synergy was defined as a ≥2-Log_10_ reduction in CFU/mL at 24 hours for antibiotic combination compared with the most active single agent, and a ≥2-Log_10_ reduction relative to the inoculum. Additivity was defined as a ≥1-Log_10_ but <2-Log_10_ mean reduction by the combination compared to most effective monotherapy. Indifference was assigned when the combination resulted in <1-Log_10_ reduction and <2-Log_10_ increase in CFU/mL compared to the individual drugs. Antagonism was defined as a ≥2-Log_10_ increase in CFU/mL at 24 hours for the antibiotic combination relative to the most active single antibiotic.

### Generation and Analysis of Mutants With Reduced FDC Susceptibility

During E-test MIC analysis, the *S. marcescens* strain K3146 produced spontaneous mutants with higher MIC values to FDC. These colonies were isolated and their MICs to both antibiotics were determined. Genomic DNA was extracted using a kit (Qiagen Gentra Puregene) following the manufacturer's specifications. Long (Oxford Nanopore) and short read (Illumina) sequencing was performed on the parent strain to generate a reference genome and the genomes of mutant strains were obtained using Illumina generated (2 × 151 base pair pair-end read data) sequencing (SeqCenter, Inc.). Genomes were analyzed for variants using BreSeq software (version 0.38.1).^38^ Each strain harbored a single mutation, except strain 6020 which had 2 mutations (see text below).

### Statistical Analysis

All statistical analyses was performed using Graphpad Prism software.

## Results

### Synergy Between FDC and PB Against XDR *P. aeruginosa* Outbreak Isolates

**Figure 1.**
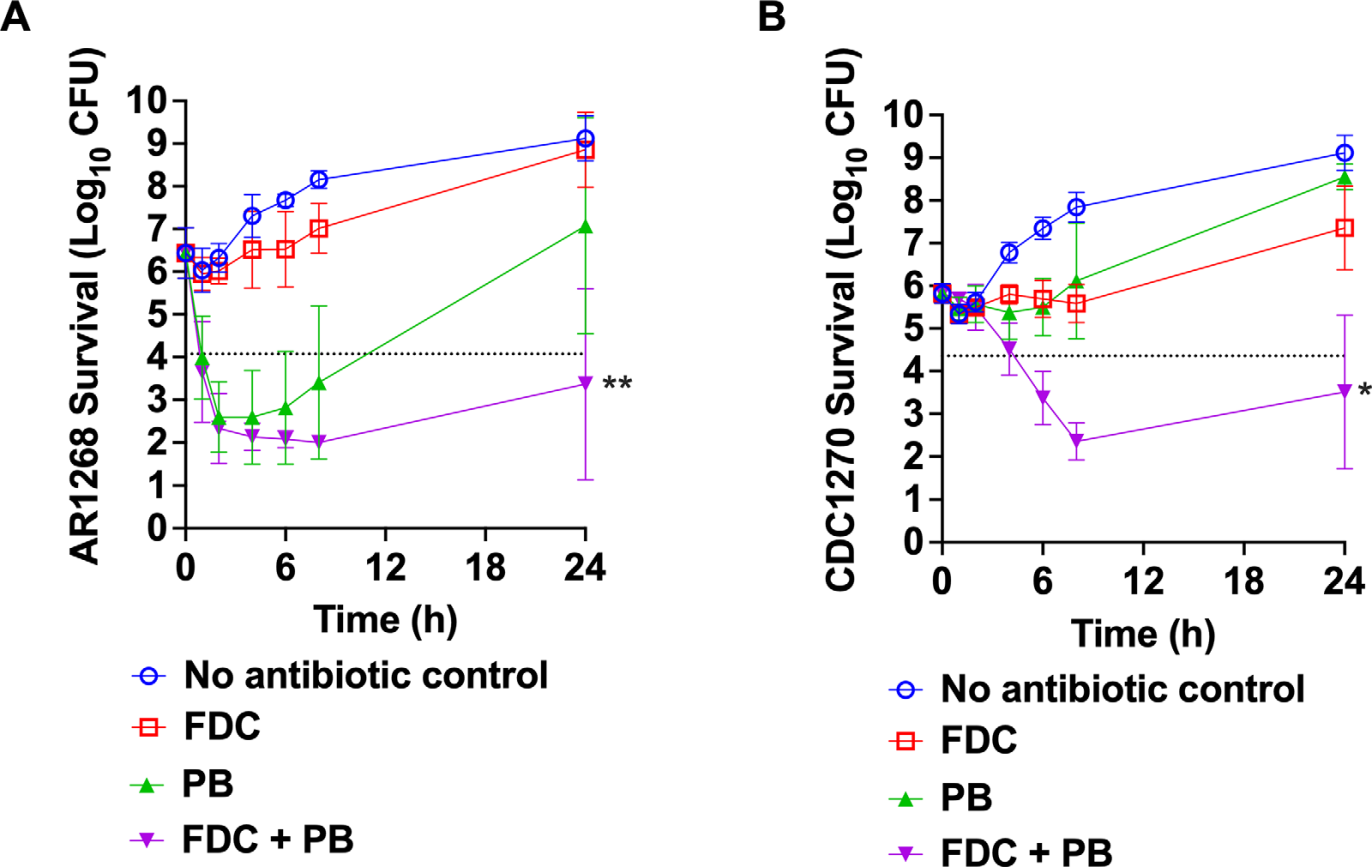
**Time-kill experiments evaluating cefiderocol and polymyxin B versus XDR outbreak isolates of *P.* *aeruginosa*.** Bacteria were exposed to cefiderocol (FDC) at 2-times MIC values and polymyxin B (PB) at one third-times MIC values which varied by isolate. (**A**) Isolate AR1268. (**B**) AR1270. Mean and SD are shown (*n* = 4). In both cases, a synergistic effect was measured. Values below the *arrow line* indicate synergy at 24 hours. Asterisks indicate significant differences of FDC + PB compared to the second most effective treatment, *P < 0.05, **P < 0.01.

Time-kill assays were performed on two *P. aeruginosa* isolates (AR-1268 and CDC1270) from the 2023 keratitis outbreak obtained from the CDC. Antibiotic concentrations were selected based on MICs determined by E-test in preliminary studies: FDC at 2 times MIC and PB at 0.33 times MIC. At these low concentrations, the drugs individually have poor in vitro efficacy. Results demonstrated synergistic activity of the FDC-PB combination against both XDR isolates (Fig. 1). The differences between the combination and the next closest group were significantly different for both strains (*P* = 0.0230 for AR-1268 and *P* = 0.0152 for CDC1270). Synergy was defined as a ≥2-log_10_ reduction in CFU/mL at 24 hours for the combination compared to the most active single agent and to the starting inoculum.^35^ These results promoted higher throughput testing using the E-test-based synergy method.^35^

### Epsilometer-Based Susceptibility and Synergy Testing of *P. aeruginosa* Keratitis Isolates

**Figure 2.**
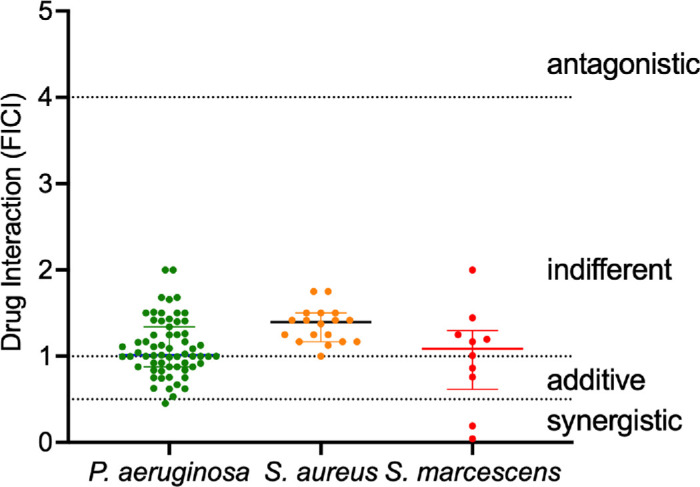
**Efficacy of cefiderocol and polymyxin B combination on keratitis bacterial keratitis isolates.** FICI were calculated for bacterial keratitis isolates using the crossed epsilometer method of synergy testing. Medians and interquartile ranges of FICI values for individual isolates are shown.

Using E-tests, 63 *P. aeruginosa* keratitis isolates were tested for susceptibility to FDC and PB alone and in combination. All isolates were susceptible to FDC (MIC_90_ = 0.38 µg/mL; CLSI breakpoint ≤4 µg/mL). PB MICs ranged from 1.5 to 4 µg/mL, with an MIC_90_ of 4 µg/mL, which was the resistance threshold (≥4 µg/mL). When tested in combination, MIC_90_ values for both antibiotics were reduced. The median FICI was 1.012 (range = 0.0454–2.0), indicating primary additive or indifferent interactions (Table 1; Fig. 2). Only one isolate exhibited synergy (FICI < 0.5), and just over 40% showed additive effects. Three outbreak isolates (AR-1268, AR-1269, and CDC1270) were also tested by E-test. AR-1268 and AR-1269 showed additive interactions (FICI = 0.788 and 0.668, respectively; FICI = 1.30; *n* ≥ 3).

**Table 1.** MIC and FICI for Keratitis Isolates With Cefiderocol and Polymyxin B

Species	MIC_90_ Cefiderocol, µg/mL	MIC_90_ Polymyxin B, µg/mL	MIC_90_ Cefiderocol in Combination, µg/mL	MIC_90_ Polymyxin B in Combination, µg/mL	FICI, Median (95% CI)
*P. aeruginosa* (*n* = 63)	0.38	4	0.125	3	1.012 (0.995–1.126)
*S. aureus* (*n* = 18)	256	24	192	16	1.396 (1.167–1.500)
*S. marcescens* (*n* = 10)	0.094	1024	0.023	256	1.087 (0.191–1.446)

95% CI, 95% confidence interval.

### Synergy Testing of *S. marcescens* and *S. aureus* Keratitis Isolates

To test broader applicability, synergy testing was extended to keratitis isolates of *S. marcescens* and *S. aureus*. *S. marcescens* was highly susceptible to FDC with an MIC_90_ of 0.094, and 8 of 20 isolates exhibiting MICs below the lowest E-test gradient (0.016 µg/mL; see Fig. 2; Table 1). By contrast, PB susceptibility was low (MIC_90_ = 1024 µg/mL). *S. aureus* keratitis isolates were resistant to FDC, consistent with prior reports with ocular isolates.^22^

Combination testing showed reduced MIC_90_ values for both species (see Table 1). However, FICI calculations were limited by extreme MIC values that were too low for FDC in isolates with MIC values below 0.016 µg/mL and too high for PB values in isolates above the 1024 µg/mL gradient. Among isolates with interpretable results, median FICI values indicated indifference: 1.087 for *S. marcescens* and 1.396 for *S. aureus*. Notably, two *S. marcescens* isolates (20%) demonstrated synergy (FICI = 0.191 and 0.044), and two others showed additive effects (FICI = 0.758 and 0.862; see Fig. 2; Table 1).

### Synergy Differs Between Sheen-Positive and Sheen-Negative *P. aeruginosa* Isolates

**Figure 3.**
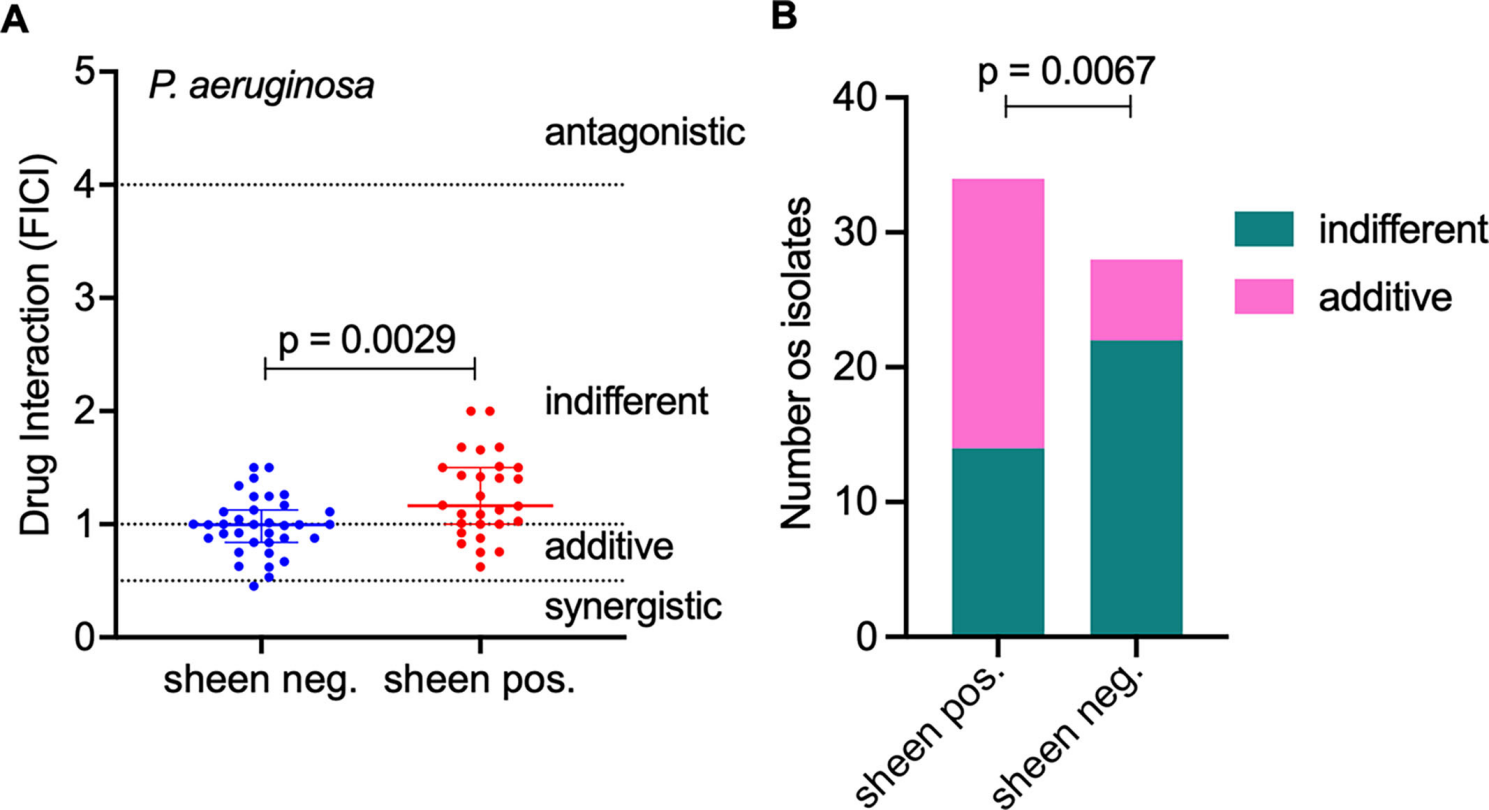
**Impact of isolate sheen status on cefiderocol-polymyxin B combined efficacy.** (**A**) Medians and interquartile ranges of FICI values for individual sheen negative and positive *P. aeruginosa* keratitis isolates is shown. A Mann-Whitney *U* test was used for comparison of medians. (**B**) Cefiderocol and polymyxin B are more likely to have an additive effect on sheen negative isolates. The Chi-square was the test used to compare the groups.

Sheen-positive *P. aeruginosa* isolates account for approximately 20% of keratitis cases and appear to be increasing in prevalence.^31^^,^^32^ The sheen-phenotype is typically (>95%) conferred by mutations in the *lasR* quorum-sensing regulator gene.^31^^,^^32^ These mutations have been linked to altered clinical outcomes in keratitis.^31^^,^^32^ To assess the impact of this phenotype on antibiotic interactions, we enriched our test set with sheen-positive isolates (44.4% of the total). Four of these have previously had their *lasR* gene sequenced and each had a mutation (one frame shift, one missense, and two with insertion elements).^32^ As shown in Figure 3A and Table 2, the median FICI was significantly higher in sheen-positive isolates (1.164) compared with sheen-negative strains (0.9950; *P* = 0.0029, Mann-Whitney *U* test). Additive interactions between FDC and PB were observed in 58.8% of sheen-negative isolates but only 21.4% of sheen-positive isolates (*P* = 0.0067; Fig. 3B). A similar trend was observed in strain PA14 (sheen-negative) and an isogenic *lasR* deletion mutant (sheen-positive), with median FICI values of 0.917 and 1.681, respectively (*n* = 3). Although not massive differences, these suggest that mutation of *lasR* affects the efficacy of the drug combination.

**Table 2.** Comparison of Sheen Positive and Negative *P. aeruginosa* Keratitis Isolates

Species^a^	MIC_90_ Cefiderocol, µg/mL	MIC_90_ Polymyxin B, µg/mL	MIC_90_ Cefiderocol in Combination, µg/mL	MIC_90_ Polymyxin B in Combination, µg/mL	Mean (SD) FIC Index
*P. aeruginosa* sheen negative (*n* = 35/35)	0.38	3	0.094	2	0.986 (0.254)
*P. aeruginosa* sheen positive (*n* = 29/28)	0.25	4	0.125	3	1.245 (0.365)

a For n-values the first number indicates n-value for MIC_90_, the second number indicates the n-value for FICI determination, which was limited by extremes in susceptibility.

### Comparison of CAZ and FDC in Combination With PB

To evaluate the role of FDC’s iron-binding domain in synergy, we compared its interactions with PB to that of CAZ, a structurally similar β-lactam lacking this domain. Median FICI values were not significantly different between the two combinations (1.476 for FDC versus 1.667 for CAZ; *P* = 0.4545, *n* = 6), suggesting the iron-binding domain may not be the primary driver of synergy in this context.

### Impact of Reduced FDC Susceptibility in *S. marcescens* Mutants

**Figure 4.**
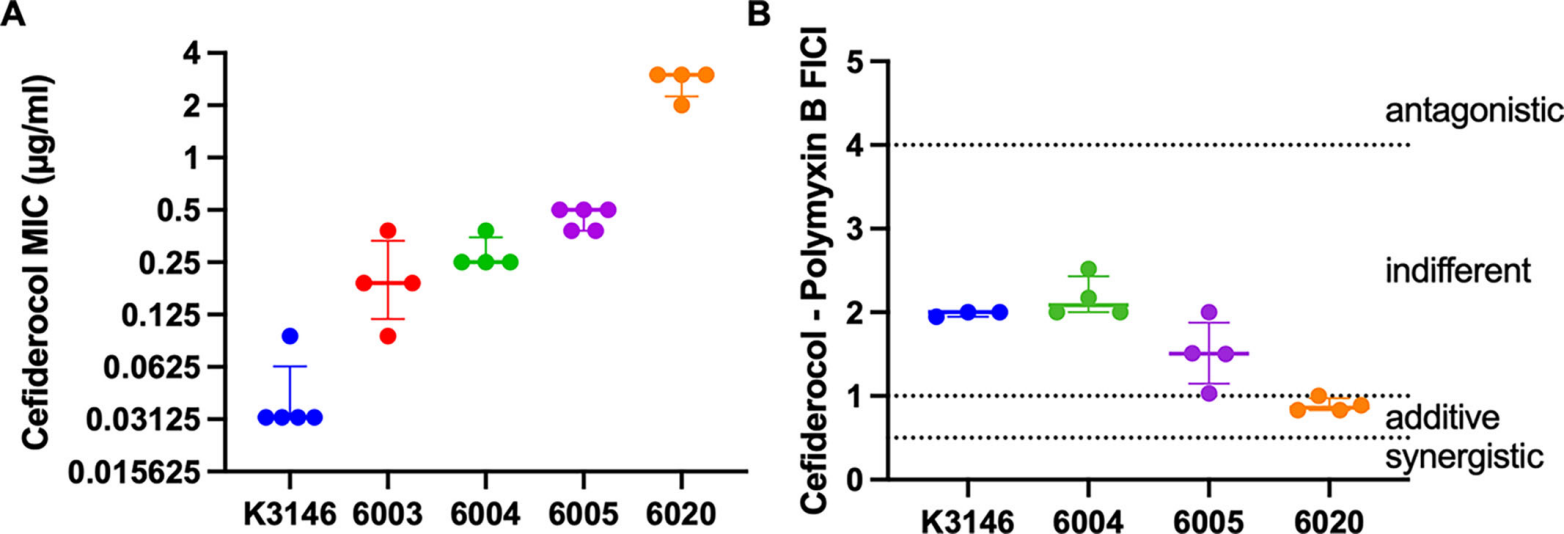
***S***. ***marcescens*** **strain K3146 derived mutants with reduced susceptibility to cefiderocol have increased relative susceptibility to the combination of polymyxin B and cefiderocol**. (**A**) Mutants were isolated during cefiderocol MIC testing using keratitis isolate K3146. The 6020 strain was isolated during testing of 6004. Repeat independent MIC values are shown. (**B**) The FICI for polymyxin B and cefiderocol is shown. Medians and interquartile ranges are shown for both panels. Epsilometer based assays were used for both analyses.

To investigate whether reduced FDC susceptibility alters drug interaction profiles with PB, we analyzed mutants of the *S. marcescens* strain K3146 that emerged spontaneously during E-test assays. The wild-type K3146 strain had a FDC MIC of 0.032 µg/mL, whereas mutants had MICs ranging from 0.190 to 0.50 µg/mL representing a 5.9 to 15.6-fold increase (Fig. 4A; Table 3).

**Table 3.** Cefiderocol Resistance Mechanisms in *S. marcescens*

Strain Number	Mutation	Change	Gene	Cefiderocol MIC, µg/mL^a^	Fold Increase Over WT	PolyPhen-2 Score^b^
K3146, WT	n/a	n/a	n/a	0.032	n/a	n/a
CMS6003	C to T	T149I	*baeS*	0.19	5.9	0.019 Benign
CMS6004	T to G	V28G	*baeS*	0.25	7.8	1.0 Probably damaging
CMS6005	A to T	Truncation after K97	*fepA*	0.5	15.6	n/a
CMS6020	G to C, T to G	G630R V28G	*fepA* *baeS*	3.0	93.8	0.912 Possibly damaging

WT, wild type.

a Median, *n* = 4 to 5.

b Prediction on whether the mutation is deleterious to protein function. Maximum score 1.0 predicting loss of function.

Whole-genome sequencing revealed single mutations in each mutant. These were in either the two-component sensor kinase gene *baeS* or the ligand-gated channel gene *fepA*. When testing the mutant CMS6004 that has a V28G change in BaeS, a variant with a higher MIC was found (CMS6020). This strain had an MIC of 3.0 µg/mL, 90-fold higher than the wild type. CMS6020 had a second mutation that mapped to the *fepA* gene (see Table 3). Taken together, the two independent mutations in both *baeS* and *fepA* leading to reduced FDC susceptibility supports a role for these genes in FDC susceptibility.

PolyPhen-2 analysis predicted that BaeS V28G and FepA G630R mutations could be damaging, whereas the BaeS T149I mutation was predicted to be benign, consistent with its more modest impact on the FDC MIC. A premature stop codon in fepA (CMS6005) likely results in a non-functional protein, although this could not be evaluated by PolyPhen-2.

Despite increased FDC MICs, PB susceptibility remained unchanged (MICs = 256–384 µg/mL). FICI values were largely stable across mutants, except for CMS6020, which showed a shift from indifference to additivity (median FICI = 0.89 vs. 1.98 for wild-type K3146; see Fig. 3B; Table 3), suggesting that reduced FDC susceptibility did not generate antagonistic FICI values.

## Discussion

This study investigated whether FDC and PB have a positive, neutral, or antagonistic interaction based on combined antimicrobial activity against bacterial keratitis isolates. The major goal was to identify improved therapeutic strategies for treating highly antibiotic-resistant infections, such as those caused by *P. aeruginosa* strains implicated in the 2023 artificial tears keratitis outbreak. A combination approach was taken based on several factors: (1) it is common in empiric treatment of bacterial keratitis; (2) the potential to broaden antimicrobial coverage; (3) the ability to reduce the development of resistance when both compounds are active; and (4) the possibility to provide synergistic enhancement of antimicrobial efficacy.^7^^,^^39^

FDC and colistin (polymyxin E) were previously reported to be effective against the XDR *P. aeruginosa* outbreak strain.^19^ Because PB is frequently used for ocular infections and shares a similar mechanism of action with colistin, it was selected for the study as a more clinically relevant alternative for topical ocular use. PB has a long history of use for treatment of ocular infections in clinical practice and experimental settings. POLYTRIM, a topical formulation of PB sulfate and trimethoprim, is widely used for ocular infections due to its broad-spectrum antimicrobial activity favorable safety and tolerable profile.^40^ The Ocular TRUST study recommended POLYTRIM in combination with either an aminoglycoside or a fourth generation fluroquinolone to enhance coverage for empiric treatment of bacterial keratitis.^41^ Other combinations, such as PB with neomycin, and 3-component formulations including dexamethasone and gramicidin, have been widely used and evaluated in clinical trials.^42^^–^^44^ More recent experimental studies have explored the combination of PB, trimethoprim, and rifampin, demonstrating synergy and reduced resistance development.^24^^–^^26^

Beyond enhancing the activity of other antimicrobials,^30^ a potential benefit of PB is that it binds to Gram-negative bacterial lipopolysaccharide (LPS), also known as endotoxin. This use may reduce LPS-induced inflammation in the eye^45^ and other tissues.^46^^–^^49^ However, its clinical utility may be limited by poor corneal and anterior chamber penetration due to its large polypeptide structure.^42^

FDC, a siderophore-cephalosporin that exploits bacterial iron uptake pathways to enhance intracellular delivery, allowing it to overcome permeability barriers in Gram-negative bacteria.^50^ Although not yet approved for topical ophthalmic use, FDC has demonstrated bactericidal activity in a rabbit model of keratitis against the XDR *P. aeruginosa* outbreak strain.^21^^,^^36^ It also exhibited low MIC_90_ values against Gram-negative ocular pathogens, including *P. aeruginosa* keratitis and endophthalmitis isolates in vitro.^21^^,^^22^

The primary result of this study is that FDC and PB demonstrated synergistic activity against two XDR *P. aeruginosa* isolates from the 2023 outbreak, as determined by time-kill analysis. E-tests were also used, allowing scalability, but lacking dynamic interactions that can be observed by the time-kill approach. When tested using the E-test method, two of three XDR isolates exhibited additive interactions. Although the two methods differ in sensitivity and resolution, both support a trend toward enhanced efficacy when the antibiotics were used in combination. Variability among isolates was also noted and is very likely influenced by bacterial genotypes, which can vary widely among *P. aeruginosa* isolates.^51^ An additional consideration is that whereas the MIC_90_ of the tested *P. aeruginosa* strains to PB was 4 µg/mL, which is considered resistant, this breakpoint is not based on topical treatment, and higher tissue concentrations of antibiotics are typically achievable with topical applications.

Among standard (not-XDR) strains of *P. aeruginosa*, *S. marcescens*, and *S. aureus*, no antagonistic interactions were observed. Additive or synergistic interactions were identified in 41% of *P. aeruginosa* and 40% of *S. marcescens* isolates. Although neither drug is typically effective against *S. aureus*, a reduction in MIC_90_ values was observed when the drugs were combined, although FICI values did not meet the threshold for additivity. Clearly, this combination would not be used for empiric treatment of keratitis or treatment of keratitis caused by Gram-positive bacteria.

A notable finding was the reduced responsiveness of sheen-positive *P. aeruginosa* isolates to the combination therapy. Sheen-negative isolates had a four-fold reduction in FDC MIC_90_ when combined with PB, compared with only a two-fold reduction measured with sheen-positive strains. The difference may be attributed to downregulation of iron uptake pathways in LasR-deficient strains, which could impair FDC uptake.^52^ Although the *lasR* locus was not sequenced in this study, prior work has shown that the vast majority (43 of 44) of sheen-positive keratitis isolate harbor *lasR* mutation,^31^^,^^32^ and that a defined *lasR* deletion mutant mirrored the observed trend. Among the arbitrarily chosen sheen-positive isolates used in the study, four were previously characterized and each had a mutation in the *lasR* gene.^32^ Together supporting that the sheen positive isolates had defects in the LasR-regulated quorum sensing system.

To assess whether FDC’s siderophore-mediated uptake contributes to synergy, we compared its interaction with PB to that of CAZ, a structurally similar cephalosporin lacking the iron-binding moiety. Comparable FICI values suggest that bacterial iron-scavenging mechanisms are not essential for the observed interaction with PB. This may be expected, as PB can improve the permeability of bacterial membranes.

Whereas clinical resistance to FDC by *S. marcescens* has been reported,^53^ the genetic basis has remained undefined. In this study, we identified mutations in two genes, *baeS* and *fepA*, that independently conferred reduced FDC susceptibility. The double mutant exhibited the highest MIC, indicating an additive effect and suggesting that these genes enhance FDC susceptibility through independent mechanisms.

The *baeS* gene codes for a sensor kinase involved in the bacterial envelope stress response and is part of the BaeSR two-component regulatory system.^54^ Mutations in *baeS* have been linked to reduced FDC susceptibility in *Acinetobacter baumannii* and *Klebsiella pneumonia* likely through upregulation of efflux pumps.^55^^–^^57^ In the *S. marcescens* strain K3146, the *baeSR* operon is located adjacent to the *mdtBCD* multidrug efflux operon, suggesting a similar resistance mechanism that will have to be evaluated in future studies.

The *fepA* gene codes for an outer membrane receptor for siderophore uptake. Mutations in *fepA* and its orthologs (e.g. *pirA* in *P. aeruginosa*) have been linked to reduced FDC susceptibility in multiple Gram-negative species.^58^^–^^62^ Our findings suggest that *S. marcescens* uses conserved FDC resistance mechanisms and that these mutations do not significantly alter the interaction profile with PB. Notably, the double mutant exhibited a shift from indifference to additivity, suggesting that the combination therapy may retain efficacy even in the context of emerging resistance.

Our study demonstrates in vitro synergy between FDC and PB against XDR *P. aeruginosa* at concentrations below the MIC of both drugs. These findings raise important clinical considerations. Notably, it is possible to deliver both FDC and PB at concentrations higher than the MIC to the corneal surface. For example, the commercially available formulation of PB and trimethoprim (POLYTRIM) contains PB at 1 mg/mL, which far exceeds concentrations used in this study. Furthermore, we previously demonstrated the safety and efficacy of topical FDC at 50 mg/mL in a rabbit model of XDR *Pseudomonas* keratitis. Given the ability to deliver these drugs at standard therapeutic concentrations, combination therapy may be particularly beneficial and most appropriate in severe or recalcitrant cases of *Pseudomonas* keratitis. It is possible that drug penetration into the corneal stroma is reduced in the setting of a severe ulcer, making the observed synergistic effects even more clinically relevant. Further animal and clinical studies are warranted to evaluate the therapeutic potential of this antimicrobial strategy.

## References

[1] Green M, Apel A, Stapleton F. Risk factors and causative organisms in microbial keratitis. *Cornea*. 2008; 27: 22–27. 18245962 10.1097/ICO.0b013e318156caf2

[2] Enzor R, Bowers EMR, Perzia B, et al. Comparison of clinical features and treatment outcomes of Pseudomonas aeruginosa keratitis in contact lens and non-contact lens wearers. *Am J Ophthalmol*. 2021; 227: 1–11. 33657419 10.1016/j.ajo.2021.02.024

[3] Hilliam Y, Kaye S, Winstanley C. Pseudomonas aeruginosa and microbial keratitis. *J Med Microbiol*. 2020; 69: 3–13. 31750813 10.1099/jmm.0.001110

[4] Keay L, Edwards K, Naduvilath T, et al. Microbial keratitis predisposing factors and morbidity. *Ophthalmology*. 2006; 113: 109–116. 16360210 10.1016/j.ophtha.2005.08.013

[5] Kowalski RP, Nayyar SV, Romanowski EG, et al. The prevalence of bacteria, fungi, viruses, and acanthamoeba from 3,004 cases of keratitis, endophthalmitis, and conjunctivitis. *Eye Contact Lens*. 2020; 46: 265–268. 31373904 10.1097/ICL.0000000000000642

[6] Shekhawat NS, Hall LN, Sulewski ME Jr., et al. Corneal culture and antibiotic susceptibility results for microbial keratitis in the mid-Atlantic region of the United States, 2016 to 2020. *Eye Contact Lens*. 2023; 49: 267–274. 37166232 10.1097/ICL.0000000000000993 PMC10330016

[7] Lin A, Rhee MK, Akpek EK, et al. Bacterial keratitis preferred practice pattern. *Ophthalmology*. 2019; 126: P1–P55. 30366799 10.1016/j.ophtha.2018.10.018

[8] Willcox MD . Review of resistance of ocular isolates of Pseudomonas aeruginosa and staphylococci from keratitis to ciprofloxacin, gentamicin and cephalosporins. *Clin Exp Optom*. 2011; 94: 161–168. 21083760 10.1111/j.1444-0938.2010.00536.x

[9] Bispo PJM, Sahm DF, Asbell PA. A systematic review of multi-decade antibiotic resistance data for ocular bacterial pathogens in the United States. *Ophthalmol Ther*. 2022; 11: 503–520. 35113406 10.1007/s40123-021-00449-9 PMC8927494

[10] Sahoo S, Alluri H, Mitra S, et al. Multidrug-resistant keratitis: challenging yet manageable. *Br J Ophthalmol*. 2023; 107: 769–773. 35346947 10.1136/bjophthalmol-2021-320203

[11] Durrani AF, Faith SC, Kowalski RP, et al. Moraxella keratitis: analysis of risk factors, clinical characteristics, management, and treatment outcomes. *Am J Ophthalmol*. 2019; 197: 17–22. 30201340 10.1016/j.ajo.2018.08.055

[12] Mediero S, Boto de Los Bueis A, Spiess K, et al. Clinical and microbiological profile of infectious keratitis in an area of Madrid, Spain. *Enferm Infecc Microbiol Clin (Engl Ed)*. 2018; 36: 409–416. 28993066 10.1016/j.eimc.2017.08.002

[13] Vazirani J, Wurity S, Ali MH. Multidrug-resistant Pseudomonas aeruginosa keratitis: risk factors, clinical characteristics, and outcomes. *Ophthalmology*. 2015; 122: 2110–2114. 26189185 10.1016/j.ophtha.2015.06.007

[14] Morelli MK, Kloosterboer A, Fulton SA, et al. Investigating and treating a corneal ulcer due to extensively drug-resistant Pseudomonas aeruginosa. *Antimicrob Agents Chemother*. 2023; 67: e0027723. 37166191 10.1128/aac.00277-23 PMC10358754

[15] Velcani F, Kuo IC, Shanks RMQ, et al. Association of artificial tears with ocular and systemic infection: carbapenem-resistant Pseudomonas aeruginosa (VIM-GES-CRPA) outbreak. *Ophthalmology*. 2023; 130: 1118–1120. 37452816 10.1016/j.ophtha.2023.06.003 PMC11182435

[16] Grossman MK, Rankin DA, Maloney M, et al. Extensively drug-resistant Pseudomonas aeruginosa outbreak associated with artificial tears. *Clin Infect Dis*. 2024; 79: 6–14. 38315890 10.1093/cid/ciae052 PMC11259536

[17] Sundermann AJ, Rangachar Srinivasa V, Mills EG, et al. Two artificial tears outbreak-associated cases of extensively drug-resistant Pseudomonas aeruginosa detected through whole genome sequencing-based surveillance. *J Infect Dis*. 2024; 229: 517–521. 37700467 10.1093/infdis/jiad318 PMC10873170

[18] Calvario RC, Shanks RMQ. Genome sequence generated by hybrid Nanopore-Illumina assembly of an extensively drug-resistant Pseudomonas aeruginosa strain from a keratitis outbreak. *Microbiol Resour Announc*. 2024; 13: e0118823. 38265222 10.1128/mra.01188-23 PMC10868217

[19] Centers for Disease Control and Prevention. Outbreak of extensively drug-resistant Pseudomonas aeruginosa associated with artificial tears. In: Network DvtCHA (ed), *DCHAN-00485*; 2023. Available at: https://archive.cdc.gov/www_cdc_gov/hai/outbreaks/crpa-artificial-tears.html.

[20] Price ER, McDermott D, Sherman A, et al. Canine multidrug-resistant Pseudomonas aeruginosa cases linked to human artificial tears-related outbreak. *Emerg Infect Dis*. 2024; 30: 2689–2691. 39592398 10.3201/eid3012.240085 PMC11616642

[21] Romanowski EG, Mandell JB, Jhanji V, Shanks RMQ. The efficacy of topical cefiderocol treatment of experimental extensively drug-resistant Pseudomonas aeruginosa keratitis is dependent upon the state of the corneal epithelium. *Antibiotics (Basel)*. 2024; 13: 979. 39452245 10.3390/antibiotics13100979 PMC11505324

[22] Schilling B, Hii M, Shanks HQ, et al. Efficacy of cefiderocol against endophthalmitis isolates. *Antibiotics (Basel)*. 2024; 13: 1236. 39766626 10.3390/antibiotics13121236 PMC11672573

[23] Kowalski RP, Kowalski TA, Shanks RM, Romanowski EG, Karenchak LM, Mah FS. In vitro comparison of combination and monotherapy for the empiric and optimal coverage of bacterial keratitis based on incidence of infection. *Cornea*. 2013; 32: 830–834. 23132444 10.1097/ICO.0b013e318268d6f4 PMC3568252

[24] Chojnacki M, Philbrick A, Scherzi T, Pecora N, Dunman PM, Wozniak RAF. A novel, broad-spectrum antimicrobial combination for the treatment of Pseudomonas aeruginosa corneal infections. *Antimicrob Agents Chemother*. 2019; 63: e00777–19. 31332071 10.1128/AAC.00777-19 PMC6761539

[25] Chojnacki M, Philbrick A, Wucher B, et al. Development of a broad-spectrum antimicrobial combination for the treatment of Staphylococcus aureus and Pseudomonas aeruginosa corneal infections. *Antimicrob Agents Chemother*. 2018; 63: e01929–18. 30420484 10.1128/AAC.01929-18 PMC6325218

[26] Mei JA, Johnson W, Kinn B, et al. Antimicrobial activity of a triple antibiotic combination toward ocular Pseudomonas aeruginosa clinical isolates. *Transl Vis Sci Technol*. 2022; 11: 26. 10.1167/tvst.11.5.26 PMC9145016 35612831

[27] Gowtham L, Wozniak RAF, Dunman PM, Sheba E, Garg P, Joseph J. Efficacy of a novel antibiotic drug combination toward multidrug-resistant ocular pathogens. *Cornea*. 2024; 43: 1044–1048. 38537125 10.1097/ICO.0000000000003528

[28] Itoi M, Willcox MDP. Combination effect of levofloxacin and cefmenoxime against ocular isolates of Pseudomonas aeruginosa. *Cont Lens Anterior Eye*. 2024; 47: 102311. 39306575 10.1016/j.clae.2024.102311

[29] Kimura Y, Matsunaga H, Vaara M. Polymyxin B octapeptide and polymyxin B heptapeptide are potent outer membrane permeability-increasing agents. *J Antibiot (Tokyo)*. 1992; 45: 742–749. 1624376 10.7164/antibiotics.45.742

[30] Wesseling CMJ, Martin NI. Synergy by perturbing the Gram-negative outer membrane: opening the door for gram-positive specific antibiotics. *ACS Infect Dis*. 2022; 8: 1731–1757. 35946799 10.1021/acsinfecdis.2c00193 PMC9469101

[31] Hammond JH, Hebert WP, Naimie A, et al. Environmentally endemic Pseudomonas aeruginosa strains with mutations in lasR are associated with increased disease severity in corneal ulcers. *mSphere*. 2016; 1: e00140–16. 27631025 10.1128/mSphere.00140-16 PMC5014915

[32] Shanks RMQ, Atta S, Stella NA, et al. A rise in the frequency of lasR mutant Pseudomonas aeruginosa among keratitis isolates between 1993 and 2021. *Front Cell Infect Microbiol*. 2023; 13: 1286842. 38029269 10.3389/fcimb.2023.1286842 PMC10651084

[33] Hoffman LR, Richardson AR, Houston LS, et al. Nutrient availability as a mechanism for selection of antibiotic tolerant Pseudomonas aeruginosa within the CF airway. *PLoS Pathog*. 2010; 6: e1000712. 20072604 10.1371/journal.ppat.1000712 PMC2795201

[34] LaFayette SL, Houle D, Beaudoin T, et al. Cystic fibrosis-adapted Pseudomonas aeruginosa quorum sensing lasR mutants cause hyperinflammatory responses. *Sci Adv*. 2015; 1: e1500199. 26457326 10.1126/sciadv.1500199 PMC4597794

[35] White RL, Burgess DS, Manduru M, Bosso JA. Comparison of three different in vitro methods of detecting synergy: time-kill, checkerboard, and E test. *Antimicrob Agents Chemother*. 1996; 40: 1914–1918. 8843303 10.1128/aac.40.8.1914 PMC163439

[36] Romanowski EG, Mumper SM, Shanks HQ, et al. Cefiderocol is an effective topical monotherapy for experimental extensively drug-resistant Pseudomonas aeruginosa keratitis. *Ophthalmol Sci*. 2024; 4: 100452. 38560275 10.1016/j.xops.2023.100452 PMC10973669

[37] Simner PJ, Patel R. Cefiderocol antimicrobial susceptibility testing considerations: the Achilles' heel of the Trojan horse? *J Clin Microbiol*. 2020; 59: e00951–20. 32727829 10.1128/JCM.00951-20 PMC7771437

[38] Deatherage DE, Barrick JE. Identification of mutations in laboratory-evolved microbes from next-generation sequencing data using breseq. *Methods Mol Biol*. 2014; 1151: 165–188. 24838886 10.1007/978-1-4939-0554-6_12 PMC4239701

[39] Coates ARM, Hu Y, Holt J, Yeh P. Antibiotic combination therapy against resistant bacterial infections: synergy, rejuvenation and resistance reduction. *Expert Rev Anti Infect Ther*. 2020; 18: 5–15. 31847614 10.1080/14787210.2020.1705155

[40] Wagner RS . Results of a survey of children with acute bacterial conjunctivitis treated with trimethoprim-polymyxin B ophthalmic solution. *Clin Ther*. 1995; 17: 875–881. 8595639 10.1016/0149-2918(95)80065-4

[41] Asbell PA, Colby KA, Deng S, et al. Ocular TRUST: nationwide antimicrobial susceptibility patterns in ocular isolates. *Am J Ophthalmol*. 2008; 145: 951–958. 18374299 10.1016/j.ajo.2008.01.025

[42] Robert PY, Adenis JP. Comparative review of topical ophthalmic antibacterial preparations. *Drugs*. 2001; 61: 175–185. 11270936 10.2165/00003495-200161020-00003

[43] Bosscha MI, van Dissel JT, Kuijper EJ, Swart W, Jager MJ. The efficacy and safety of topical polymyxin B, neomycin and gramicidin for treatment of presumed bacterial corneal ulceration. *Br J Ophthalmol*. 2004; 88: 25–28. 14693766 10.1136/bjo.88.1.25 PMC1771930

[44] Tromans A . Physiology: joint approach. *Nature*. 2004; 431: 921. 15496908 10.1038/431921a

[45] Morck DW, Holland SP, Ceri H, et al. Use of polymyxin as an endotoxin blocker in the prevention of diffuse lamellar keratitis in an animal model. *J Refract Surg*. 2005; 21: 152–157. 15796220 10.3928/1081-597X-20050301-10

[46] Corrigan JJ Jr., Bell BM. Comparison between the polymyxins and gentamicin in preventing endotoxin-induced intravascular coagulation and leukopenia. *Infect Immun*. 1971; 4: 563–566. 4343409 10.1128/iai.4.5.563-566.1971 PMC416353

[47] Cavaillon JM, Haeffner-Cavaillon N. Polymyxin-B inhibition of LPS-induced interleukin-1 secretion by human monocytes is dependent upon the LPS origin. *Mol Immunol*. 1986; 23: 965–969. 3023974 10.1016/0161-5890(86)90127-6

[48] Ebihara I, Nakamura T, Shimada N, Shoji H, Koide H. Effect of hemoperfusion with polymyxin B-immobilized fiber on plasma endothelin-1 and endothelin-1 mRNA in monocytes from patients with sepsis. *Am J Kidney Dis*. 1998; 32: 953–961. 9856510 10.1016/s0272-6386(98)70069-1

[49] Barton MH, Parviainen A, Norton N. Polymyxin B protects horses against induced endotoxaemia in vivo. *Equine Vet J*. 2004; 36: 397–401. 15253079 10.2746/0425164044868350

[50] Choi JJ, McCarthy MW. Cefiderocol: a novel siderophore cephalosporin. *Expert Opin Investig Drugs*. 2018; 27: 193–197. 10.1080/13543784.2018.1426745 29318906

[51] Kung VL, Ozer EA, Hauser AR. The accessory genome of Pseudomonas aeruginosa. *Microbiol Mol Biol Rev*. 2010; 74: 621–641. 21119020 10.1128/MMBR.00027-10 PMC3008168

[52] Stintzi A, Evans K, Meyer JM, Poole K. Quorum-sensing and siderophore biosynthesis in Pseudomonas aeruginosa: lasR/lasI mutants exhibit reduced pyoverdine biosynthesis. *FEMS Microbiol Lett*. 1998; 166: 341–345. 9770291 10.1111/j.1574-6968.1998.tb13910.x

[53] Kohira N, West J, Ito A, et al. In vitro antimicrobial activity of a siderophore cephalosporin, S-649266, against Enterobacteriaceae clinical isolates, including carbapenem-resistant strains. *Antimicrob Agents Chemother*. 2016; 60: 729–734. 26574013 10.1128/AAC.01695-15 PMC4750680

[54] Macritchie DM, Raivio TL. Envelope stress responses. *EcoSal Plus*. 2009; 3: 7. 10.1128/ecosalplus.5.4.7 26443759

[55] Simner PJ, Beisken S, Bergman Y, Ante M, Posch AE, Tamma PD. Defining baseline mechanisms of cefiderocol resistance in the Enterobacterales. *Microb Drug Resist*. 2022; 28: 161–170. 34619049 10.1089/mdr.2021.0095 PMC8885434

[56] Liu X, Chang Y, Xu Q, et al. Mutation in the two-component regulator BaeSR mediates cefiderocol resistance and enhances virulence in Acinetobacter baumannii. *mSystems*. 2023; 8: e0129122. 37345941 10.1128/msystems.01291-22 PMC10469669

[57] Yang C, Wang L, Lv J, et al. Effects of different carbapenemase and siderophore production on cefiderocol susceptibility in Klebsiella pneumoniae. *Antimicrob Agents Chemother*. 2024; 68: e0101924. 39470196 10.1128/aac.01019-24 PMC11619314

[58] Daoud L, Al-Marzooq F, Moubareck CA, Ghazawi A, Collyns T. Elucidating the effect of iron acquisition systems in Klebsiella pneumoniae on susceptibility to the novel siderophore-cephalosporin cefiderocol. *PLoS One*. 2022; 17: e0277946. 36580460 10.1371/journal.pone.0277946 PMC9799297

[59] Zhang Q, Neidig N, Chu TY, et al. In vitro antibacterial activity of cefiderocol against recent multidrug-resistant carbapenem-nonsusceptible Enterobacterales isolates. *Diagn Microbiol Infect Dis*. 2022; 103: 115651. 35228130 10.1016/j.diagmicrobio.2022.115651

[60] Karakonstantis S, Rousaki M, Vassilopoulou L, Kritsotakis EI. Global prevalence of cefiderocol non-susceptibility in Enterobacterales, Pseudomonas aeruginosa, Acinetobacter baumannii, and Stenotrophomonas maltophilia: a systematic review and meta-analysis. *Clin Microbiol Infect*. 2024; 30: 178–188. 37666449 10.1016/j.cmi.2023.08.029

[61] Padovani M, Bertelli A, Corbellini S, Piccinelli G, Gurrieri F, De Francesco MA. In vitro activity of cefiderocol on multiresistant bacterial strains and genomic analysis of two cefiderocol resistant strains. *Antibiotics (Basel)*. 2023; 12: 785. 37107147 10.3390/antibiotics12040785 PMC10135176

[62] Polani R, De Francesco A, Tomolillo D, et al. Cefiderocol resistance conferred by plasmid-located ferric citrate transport system in KPC-producing Klebsiella pneumoniae. *Emerg Infect Dis*. 2025; 31: 123–124. 39714320 10.3201/eid3101.241426 PMC11682805

